# Effects of Flammable and Non-Flammable Nicotine Products on Kidney Health

**DOI:** 10.3390/medsci14030386

**Published:** 2026-07-09

**Authors:** Caterina Carollo, Alessandra Sorce, Emanuele Cirafici, Maria Elena Ciuppa, Nicola Sinatra, Giulio Geraci, Giuseppe Mulè

**Affiliations:** 1 Department of Health Promotion, Mother and Child Care, Internal and Specialistic Medicine of Excellence “G. D’Alessandro” (PROMISE), University of Palermo, 90127 Palermo, Italy; alessandra.sorce@community.unipa.it (A.S.); emanuele.cirafici@community.unipa.it (E.C.); mariaelena.ciuppa@community.unipa.it (M.E.C.); giuseppe.mule@unipa.it (G.M.); 2 Nephrology and Dialysis Unit, “Paolo Borsellino” Hospital, 91025 Marsala, Italy; sinatra.nicola@libero.it; 3 Department of Medicine and Surgery, “Kore” University of Enna, 94100 Enna, Italy; giulio.geraci@unikore.it

**Keywords:** smoking, cigarette smoking, e-cigarettes, nicotine, tobacco, heated tobacco products, oral nicotine products, CKD, kidney damage

## Abstract

**Background:** Cigarette smoking is a widely recognized risk factor for several diseases and a crucial problem in global health, as it contributes to the development and worsening of various diseases, resulting in an increase in both morbidity and mortality. One of the strategies to reduce the impact of tobacco damage is based on the transition from flammable to non-flammable nicotine products. **Objectives:** The aim of this narrative review is to summarize the effects of traditional cigarette smoking and non-combustible nicotine products on kidney function. **Conclusions:** The evidence shows that both combustible and non-combustible nicotine products have harmful effects on kidney health and may promote the onset and progression of kidney dysfunction, leading to chronic kidney disease (CKD).

## 1. Introduction

Cigarette smoking is a widely recognized leading risk factor for several diseases, such as arterial hypertension, atherosclerosis, chronic obstructive pulmonary disease (COPD), cancer, and cardiovascular and kidney diseases [1,2,3,4].

The harmful effects of cigarette smoking are mainly mediated by oxidative stress, chronic inflammation and sympathetic system activation [1,4].

An important role in cigarette smoking-mediated damage is also played by the released toxic chemicals, approximately 7000 substances, the combustion of which promotes organ damage [5].

Despite the harmful effects of smoking being widely known, the number of smokers still remains very high. According to the most recent “WHO” data, tobacco addiction causes over 8 million deaths each year; among these, more than 7 million are the result of direct smoking, while approximately 1.3 million arise from exposure to second-hand smoke (SHS). Europe is the region with the second highest prevalence of smokers both among adolescents, aged 13–15, (10.8%) and adults (25.3%) [6].

While previously cigarette smoking was mainly a male habit, currently the gap between men and women is no longer relevant (<5%) [6].

Cigarette smoking is a crucial problem in global health, as it contributes to the development and worsening of various diseases, resulting in an increase in both morbidity and mortality [2,3]. To reduce the impact of this condition, many campaigns have been implemented to raise awareness among the population, although one of the major difficulties consists of counteracting the addiction to tobacco use [2].

One of the strategies to reduce tobacco damage, which is mainly caused by combustion products, is based on the transition from combustible to non-combustible tobacco devices, including

- Electronic nicotine delivery systems (ENDSs), such as electronic cigarettes (e-cigs);
- Heated tobacco products (HTPs);
- Oral nicotine products.

These combustion-free products have been shown to provide less exposure to toxic substances than combustible cigarettes, suggesting fewer harmful effects on general health from these devices [7].

The aim of this narrative review is to summarize the effects of cigarette smoking and non-combustible nicotine products on kidney function.

## 2. Materials and Methods

This article is a narrative review. We carried out a search in PubMed, Elsevier, Oxford Academic, BioMed Central and MDPI from July 1996 to July 2025 with the following keywords: “smoking”, “cigarette smoking”, “nicotine”, “tobacco”, “e-cigarettes”, “HTP”, “oral nicotine products”, “non-combusted cigarettes” and/or “CKD”, “kidney diseases” and “renal function”.

We applied a broad range of inclusion criteria to include all articles relevant to the effects of traditional smoking and non-combustible nicotine products on kidney health.

Studies were excluded if they were clearly unrelated to the keywords previously listed, showed incorrect references, or were based on a very small sample size (fewer than 50 participants). Only English-language, peer-reviewed articles were considered, with no restriction on publication date. Both clinical (human and animal) and mechanistic (in vitro) studies were included where relevant to the topic. A total of 96 articles were identified through the search, of which 17 were included in the review after applying the inclusion/exclusion criteria described above.

## 3. Cigarette Smoking and Kidney Disease

The effects of cigarette smoking on kidney function have been studied for several years. Smoking is recognized to worsen renal function and it is a significant risk factor for the progression to kidney failure [8]. Studies show that both direct and second-hand smoke can impair kidney and glomerular function [9,10]. A link exists between exposure to SHS and CKD both in adults [11] and in children. In a cohort of 366 children with CKD (aged 1–16) SHS exposure was associated with nephrotic range proteinuria [12].

Different studies searched for a correlation between estimated glomerular filtration rate (eGFR), microalbuminuria, and serum levels of cotinine in both active and passive smokers [9,10]. Cotinine is a primary metabolite of nicotine, characterized by a long half-life (3–4 days), and is widely used as marker of exposure to tobacco [13]. The analyses show a positive correlation between serum cotinine levels and microalbuminuria in both active and passive smokers [9] and a negative correlation with the eGFR in people over 20 years old [10]. These data suggest that direct and second-hand smoking can lead to a decrease in renal function and can accelerate the progression of CKD to end-stage kidney disease (ESKD) [14,15], thus emerging as an independent risk factor for incident CKD [16].

A direct relationship between kidney functional decline and the number of packs smoked per year was observed through a dose–response and duration-dependent mechanism: longitudinal data also show that long-term cessation of smoking (≥10 years) may decrease the risk of renal failure [17,18].

Cigarette smoking is not only a risk factor for the development and progression of CKD, but also an emerging risk factor for other kidney diseases such as hypertensive and diabetic nephropathy, especially in patients with type 1 diabetes mellitus (T1DM) [19,20,21]; it may accelerate the progression of IgA nephropathy [22]; furthermore it is associated with increased endothelial dysfunction and atherosclerosis in patients with autosomal dominant polycystic kidney disease (ADPKD) [23].

Other interesting results are derived from the “Atherosclerosis Risk in Communities” (ARIC) study, a prospective observational study that examined the relationship between cigarette smoking and the risk of acute kidney injury (AKI). A total of 14,571 participants were followed over a median period of 26 years. Cigarette smoking was strongly associated with the risk of AKI in a dose–response manner. The risk of AKI decreased 10 years after quitting smoking and after 30 years it became similar to never smokers [24]. These findings suggest the benefit of smoking cessation for kidney health.

Significant information derived from renal biopsy specimens were obtained from smokers: glomerulosclerosis, arteriolar hyalinosis, interstitial fibrosis and tubular atrophy [15,25] are the most common findings. The pathophysiological mechanisms involved in smoking-related kidney damage are still unclear. The main hypothesized mechanisms include oxidative stress, platelet activation, nitric oxide depletion, chronic inflammation, direct cadmium-mediated tubular toxicity, hemodynamic alterations induced by nicotine leading to endothelial dysfunction, microvascular damage, glomerular hyperfiltration and sclerosis [10,15]. These elements lead to proteinuria, initial hyperfiltration, eGFR decline and progression of renal dysfunction. The association between cigarette smoking and renal hyperfiltration (RHF) is a widely recognized condition leading to kidney impairment [26,27]; RHF is an established key factor that mediates smoking effects on non-cardiovascular and all-cause mortality [28].

**Figure 1:**
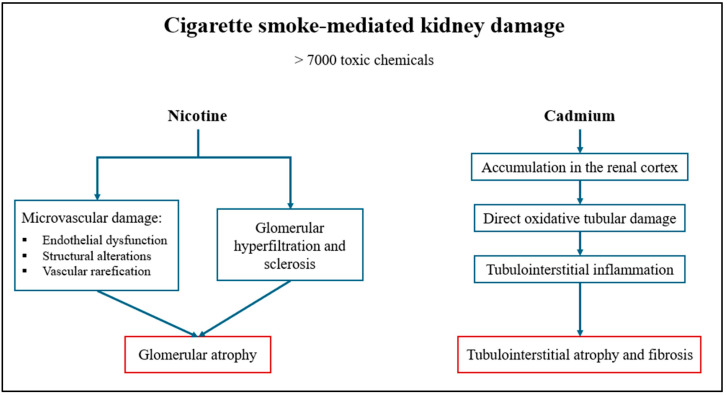
Summary of the main mechanisms potentially implicated in cigarette smoke-mediated kidney damage [15].

Chronic cadmium exposition, in turn, causes its accumulation in the renal cortex of cigarette smokers, production of reactive oxygen species (ROS) and direct oxidative tubular damage. As a consequence, tubular epithelial cells undergo functional and structural changes due to inflammatory and fibrogenic cells, with subsequent production of molecules that cause tubulo-interstitial inflammation, fibrosis and atrophy [15,29] (Figure 1).

Nicotine exerts a nephrotoxic effect, so it is a relevant link between smoking and kidney dysfunction. Nicotine induces mesangial cell proliferation and promotes fibronectin and ROS synthesis and these alterations are involved in CKD onset and progression [15,30]. Nicotine can alter systemic and renal hemodynamics through vasoconstriction, leading to endothelial dysfunction and microvascular damage; it also promotes podocyte disfunction, glomerular fibrosis and subsequent albuminuria and GFR decline [31] (Figure 1). Other nicotine effects include fibroblast activation and pro-inflammatory and pro-oxidative properties [31,32,33]. Table 1 shows the summary of the main mechanisms implicated in kidney damage.

## 4. Effects of Non-Combustible Nicotine Products on Kidney Function

In recent years there has been an exponential increase in the use of non-combustible nicotine products such as e-cig/ENDS and HTPs and oral nicotine products as alternatives to conventional cigarettes, mainly among adolescents and young adults [35]. Comparative emission analyses show that these devices appear to release fewer toxic substances than conventional cigarettes, especially HTPs, which heat tobacco without burning it, thus reducing the production of many harmful substances [36,37]. However, these devices also have harmful effects on kidney function.

E-cigarettes release several toxic molecules, including volatile organic compounds, formaldehyde, acetaldehyde, acrolein, tobacco-specific nitrosamines (TSNAs), and heavy metals such as lead (Pb) and nickel (Ni) [38] (Table 2); these substances have important nephrotoxic effects.

Furthermore, the components of e-cigs include cadmium and nicotine [39], with a relationship between elevated serum cadmium levels and e-cigarette use [40].

Cross-sectional studies suggest an association between e-cig use and kidney damage (albuminuria and a decrease in eGFR) in both adolescents and young adults [41].

HTPs and oral nicotine products have fewer but similar harmful effects due to nicotine and other toxic substances such as acrolein, benzene, and TSNAs [42,43]. The main mechanisms involved in nicotine-related kidney damage have already been described. Further effects involve renin–angiotensin–aldosterone system (RAAS) homeostasis [44], the activation of the hypothalamic–pituitary–adrenal (HPA) axis and sympathetic stimulation [45,46], podocyte damage through NLRP3 inflammasome activation and subsequent reduced expression of podocin and nephrin [47].

These data show that the long-term effects of non-combustible nicotine products on renal function are not fully understood and require further investigation.

**Table 2:** Summary of the main nephrotoxic substances related to non-combustible nicotine products [38,39,42,43].

Nicotine	Acrolein
Cadmium (Cd)	Formaldehyde
Lead (Pb)	Acetaldehyde
Nichel (Ni)	Reactive oxygen species (ROS)
Benzene	Tobacco-specific nitrosamines (TSNAs)

## 5. Molecular Patterns Involved in Smoking-Mediated Kidney Damage

**Figure 2:**
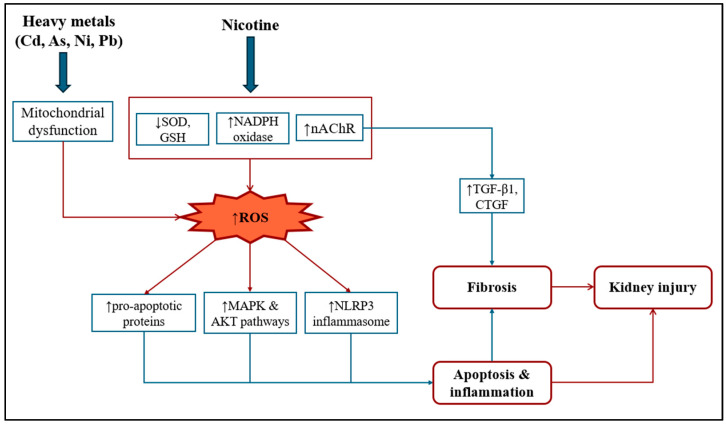
Main molecular mechanisms implicated in smoking-mediated kidney damage [29,32,38,48]. SOD: superoxide dismutase; GSH: glutathione; NADPH: nicotinamide adenine dinucleotide phosphate (reduced form); nAChR: nicotine acetylcholine receptor; TGF-β1: transforming growth factor; CTGF: connective tissue growth factor; MAPK: mitogen-activated protein kinase.

The principal nephrotoxic effects of both traditional cigarettes and non-combustible nicotine devices are largely mediated by cadmium and nicotine. These substances can damage the kidneys through various molecular mechanisms, as illustrated in Figure 2.

Although their role in renal injury is well established, other cited nephrotoxic agents deserve greater attention.

### 5.1. Nephrotoxic Effects of Heavy Metals

It is well known that exposure to heavy metals, such as lead, nickel, cadmium and arsenic, exerts nephrotoxic effects primarily due to their ability to induce mitochondrial dysfunction and generate ROS, thus leading to oxidative stress and subsequent cellular damage (Figure 2) [48,49,50,51].

In addition to oxidative stress, there are other mechanisms implied in heavy metal-mediated renal damage.

Arsenic can activate mitogen-activated protein kinases (MAPKs) in a dose-dependent manner and promote inflammation through the upregulation of nuclear factor kB (NF-kB) [48]. It appears to increase cardiovascular risk by inducing endothelial disfunction through the inhibition of endothelial nitric oxide synthase (eNOS) [48]. Moreover, arsenic may directly damage podocytes and subsequently cause albuminuria [50].

Lead nephrotoxicity involves decreased synthesis of eicosanoids, increased thromboxane B2 levels, and the phagocytosis of erythrocytes by renal cells due to enhanced phosphatidylserine externalization, thus leading to iron accumulation and subsequent oxidative damage [52]. In addition, lead has been shown to promote apoptosis in proximal tubule cells through activation of the AMPK pathway and TLR4 receptor in experimental models [52]. Other nephrotoxic mechanisms include the activation of inflammatory pathways through the upregulation of NF-kB and the impairment of calcium signalling [52].

In mouse renal cells, nickel has been shown to damage the kidney via induction of autophagy, ferroptosis (iron-dependent lipid peroxidation which leads to cell death) [53], and pyroptosis (Caspase-1-dependent inflammatory programmed cell death) via the Nrf2/NLRP3 signalling pathway in mouse renal cells [54]. Moreover, nickel promotes mitochondrial fission while inhibiting their fusion and biogenesis, resulting in mitochondrial damage and dysfunction [51].

### 5.2. Acetaldehyde, Formaldehyde and Acrolein Patterns of Renal Damage

The molecular mechanisms underlying the nephrotoxic effects of these agents are not yet fully understood due to the lack of available data.

In vitro evidence indicates that acrolein promotes activation of hypoxia-inducible factor 1-alpha (HIF-1α) signalling, thus leading to mitochondrial dysfunction and ROS production [55]. In addition, acrolein can directly inhibit aldehyde dehydrogenase 2 (ALDH2), a detoxifying enzyme whose downregulation is associated with the development of renal fibrosis and the progression of CKD [55].

It is demonstrated that formaldehyde has severe nephrotoxic effects, largely due to its ability to cause oxidative damage by impairing the anti-oxidant defence systems in renal cells [56]. It was also observed that formaldehyde exposure leads to tubule-glomerular thickening and degeneration [56].

The nephrotoxic effects of acetaldehyde are less studied; however, evidence from in vitro experiments in mouse model suggests that it can activate the integrin β1/JNK pathway, thereby promoting tubular cell apoptosis [57].

### 5.3. Summary of Clinical Evidence

The following table summarizes the main characteristics of the clinical studies cited (Table 3).

## 6. Conclusions

Smoking represents an independent risk factor for kidney damage, regardless of the presence of pre-existing chronic kidney disease. Both combustible and non-combustible nicotine products promote the onset and progression of renal dysfunction through multiple molecular mechanisms, ultimately leading to CKD and ESKD.

The evidence supports a dose–response and duration-dependent relationship between smoking exposure and kidney damage, with long-term cessation associated with a reduced—though not fully reversed—risk of renal impairment. This underscores the importance of early intervention and smoking prevention across all stages of life.

While conventional cigarettes appear to exert greater nephrotoxicity than non-combustible alternatives, the latter should not be considered safe. E-cigarettes, HTPs and oral nicotine products share key nephrotoxic substances—particularly nicotine and cadmium—and their long-term renal effects remain incompletely understood.

From a clinical perspective, smoking cessation counselling should be considered a cornerstone of nephroprotective strategies, not only by nephrologists but also by all clinicians—including general practitioners, cardiologists and internists—who encounter smokers in daily practice. Every patient who smokes is a patient at risk for kidney disease, irrespective of their current renal function.

These findings highlight the urgent need for targeted public health campaigns aimed at primary prevention of smoking, with particular attention to adolescents and young adults, among whom the use of non-combustible devices is rapidly growing.

## Figures and Tables

**Table 1:** Summary of the main nephrotoxic substances related to cigarette smoking [15,29,30,31,32,33,34].

Nicotine	Lead (Pb)
Cadmium (Cd)	Acrolein
Reactive oxygen species (ROS)	Formaldehyde
Carbon monoxide	Arsenic (As)
Polycyclic aromatic hydrocarbons (PAHs)	Tobacco-specific nitrosamines (TSNAs)

**Table 3:** Synthesis of evidence from the studies included in the review.

Reference	Population	Age	Exposure/Control Group	Objective	Findings
Hallan et al. [8]	65,589 adults from the “HUNT II” study; median follow-up: 10.3 years	20–70	Current vs. former vs. never cigarette smokers	To investigate the role of cigarette smoking in renal damage and progression to ESKD	Smoking was independently associated with increased risk of ESKD: HR = ~4–3 respectively for current/former smokers vs. never smokers.
Dülger et al. [9]	64 volunteers		Active smokers; passive smokers; controls (healthy non-smokers)	To investigate the effects of active and passive smoking on kidney function	- Urine microalbumin significantly elevated in active smokers (*p* < 0.01); - ACR increased in both active and passive smokers vs. controls (*p* < 0.01).
Fu et al. [10]	8138 adults from “NHANES” (2013–2018)	20–80	Serum cotinine (marker of smoking exposure)	To evaluate the association between smoking exposure (cotinine levels) and renal function (eGFR)	- Negative association between serum cotinine and eGFR; - Inverse relationship; stronger in ages 20–39, but weaker in those >40.
Jhee et al. [11]	131,196 never-smokers with normal kidney function from the “Korean Genome & Epidemiology Study” (2001–2014)	53 (mean age)	Frequency of SHS exposure: <3 days/week; >3 days/week; no exposure	To analyze the association between SHS exposure and the risk of CKD development among never-smokers	Higher risk of developing CKD in both SHS-exposed groups compared to the no-exposure group.
Omoloja et al. [12]	366 children with CKD from the CKiD study	1–16	SHS exposure via urine cotinine (Ucot = 1–75 ng/mL) vs. no exposure (Ucot < 1 ng/mL)	To evaluate the prevalence and effects of SHS in children with CKD	- A total of 22% were exposed to SHS; - Significantly association between SHS exposure and nephrotic-range proteinuria.
Jin et al. [17]	63,257 Chinese adults from the Singapore Chinese Health study, followed over 13.3 years	45–74 years at baseline (mean age ≈ 55.6 years)	Current vs. former vs. never cigarette smokers	To investigate the association between smoking (status, duration, intensity, and cessation) and risk of kidney failure	- Smoking was associated with increased risk of kidney failure; - Strong dose-dependent association (longer duration = higher risk); - Risk decreased after >10 years of cessation.
Lee et al. [18]	1951 patients with eGFR > 15 mL/min/1.73 m^2^ from the Korean “KNOW-CKD” study		Smoking load groups (pack-years): never-smokers; <15; 15–29; ≥30	To assess the association between smoking burden and CKD progression	- Increased risk of CKD progression with smoking load (HR: 1.09, 1.48 and 1.94 for <15, 15–29 and ≥30 pack-years); - Risk reduced with longer smoking cessation.
Yacoub et al. [20]	198 patients with CKD (stage 3–5) vs. 371 matched healthy controls (2005–2009)	Mean age ≈ 45 years	Never smokers; former regular smokers (>5 years quit); current regular smokers (smoked in the last 5 years)	To evaluate the association between cigarette smoking and CKD risk and its role on each type of renal failure	- Smoking increased CKD risk (OR 1.6); - Higher risk of CKD in current (OR 1.63) vs. no smokers; - Dose–response pattern by pack-years (16–30: OR 2.04, >30: OR 2.6); - Strong associations with hypertensive and diabetic nephropathy (respectively: OR 2.85, *p* = 0.01; OR 2.24; *p* = 0.005).
Wang et al. [22]	1239 patients with IgAN from West China Hospital of Sichuan University;	Mean age: smokers 38.6; no-smokers 33.2	Smokers (current or former) vs. non-smokers	To evaluate the impact of cigarette smoking on the progression and prognosis of IgAN	- A total of 19% of smokers vs. 11% of non-smokers reached the study endpoint (ESKD) [*p* < 0.001]; - Smoking was an independent risk factor for IgAN progression (HR = 1.58; *p* = 0.043); - Dose–response relationship: >20 pack-years increased risk by 143% compared to non-smokers; - Smoking dose was correlated negatively with eGFR (r = 0.141; *p* < 0.001) but positively with proteinuria (r = 0.096; *p* = 0.001).
Gul et al. [23]	54 ADPKD patients with preserved renal function and 45 healthy controls	ADPKD patients: 45.5 years; controls: 39 years	Smokers vs. non-smokers with ADPKD; healthy controls and smokers vs. non-smokers	To assess the role of smoking on endothelial dysfunction (ED) and subclinical atherosclerosis in ADPKD patients	- ADPKD patients had significantly lower FMD than controls; - Smokers had lower FMD than non-smokers among ADPKD patients; - Smoking independently predicted higher CIMT.
Chen et al. [24]	14,571 adults from the “ARIC” study, followed over 26 years	45–64 (mean age 55)	Smoking status, intensity, pack-years, duration and cessation: current smokers, former smokers, and never smokers	To investigate the association between cigarette smoking (its duration, intensity, and cessation) and the risk of AKI	- Smokin was strongly associated with increased risk of AK; - Risk decreased after smoking cessation.
Mickelsson et al. [26]	Two cohorts: respectively with 949 and 995 healthy participants without prior kidney disease		Active smokers vs. non-smokers	To assess the impact of tobacco use on eGFR (estimated by creatinine and cystatin C)	- Smoking was independently associated with higher eGFR based on creatinine; - Inverse association between smoking and eGFR based on cystatin C.
Maeda et al. [27]	10,118 healthy Japanese men without baseline proteinuria or renal dysfunction	44–55	Current smokers vs. past smokers vs. non-smokers	To examine the association between smoking and early renal damage (glomerular hyperfiltration and proteinuria)	Higher risk of developing glomerular hyperfiltration and proteinuria in current smokers compared to non-smokers.
Aissani et al. [28]	2064 Finnish men from the “KIHD” study, followed over 28 years	42–62	Smokers vs. non-smokers	To examine whether renal hyperfiltration mediates the relationship between smoking and mortality (all-cause, CVD, and non-CVD)	- Smokers had double the risk of all-cause mortality compared to non-smokers (HR ≈ 2.06); - Renal hyperfiltration mediated approximately 5% of the effect of smoking on non-CVD mortality (*p* = 0.016).
Rahman et al. [34]	4117 US adults from “NHANES” (2015–2016)	≥20 years	Levels of seven urinary PAHs	To investigate the relationship between urinary PAH concentrations and CKD	Urinary 2-hydroxynaphthalene was significantly associated with increased odds of CKD (OR 1.60).
Smith et al. [39]	3450 adults from Wave 1 of the “PATH” study (2013–2014)	≥18 years	Dual users of traditional cigarettes and e-cigarettes (4 subgroups: predominant smokers, daily dual users, non-daily dual users, and predominant vapers); vs. exclusive cigarette smokers and exclusive e-cig users	To examine the exposure to nicotine and tobacco-related toxicants among dual users compared to exclusive e-cig or cigarette users	Significantly higher concentrations of tobacco-specific biomarkers among daily dual users compared to non-daily users.
Lee et al. [40]	4744 adults from the “KNHANES”	Mean age: 45.7 for men and 47.3 for women	5 groups: non-smokers, e-cig non-users in former smokers, e-cig users in former smokers, e-cig non-users in cigarette smokers and e-cig users in cigarette smokers	To assess the association between blood cadmium concentration and smoking status, including e-cig use	Both conventional smoking and e-cig use were associated with significantly higher blood cadmium levels than those observed in non-smokers.

ESKD = end-stage kidney disease; HR = hazard ratio; ACR = albumin–creatinine ratio; eGFR = estimated glomerular filtration rate; CKD = chronic kidney disease; SHS = second-hand smoke; OR = odds ratio; IgAN = IgA nephropathy; ADPKD = autosomal dominant polycystic kidney disease; FMD = flow-mediated dilatation; CIMT = carotid intima-media thickness; AKI = acute kidney injury; CVD = cardiovascular disease; PAHs = polycyclic aromatic hydrocarbons.

## Data Availability

No new data were created or analyzed in this study.

## References

[1] Virdis A., Giannarelli C., Neves M.F., Taddei S., Ghiadoni L. (2010). Cigarette smoking and hypertension. Curr. Pharm. Des..

[2] Lushniak B.D., Samet J.M., Pechacek T.F., Norman L.A., Taylor P.A. The Health Consequences of Smoking—50 Years of Progress: A Report of the Surgeon General. https://stacks.cdc.gov/view/cdc/21569.

[3] Varghese J., Muntode Gharde P. (2023). A Comprehensive Review on the Impacts of Smoking on the Health of an Individual. Cureus.

[4] Ishida M., Sakai C., Kobayashi Y., Ishida T. (2024). Cigarette Smoking and Atherosclerotic Cardiovascular Disease. J. Atheroscler. Thromb..

[5] Soleimani F., Dobaradaran S., De-la-Torre G.E., Schmidt T.C., Saeedi R. (2022). Content of toxic components of cigarette, cigarette smoke vs cigarette butts: A comprehensive systematic review. Sci. Total Environ..

[6] Status of Tobacco Use in the Region. https://www.who.int/europe/news-room/fact-sheets/item/tobacco.

[7] Polosa R., Rodu B., Farsalinos K. (2025). Health effects of e-cigarettes, heated tobacco, and oral nicotine products. Intern. Emerg. Med..

[8] Hallan S.I., Orth S.R. (2011). Smoking is a risk factor in the progression to kidney failure. Kidney Int..

[9] Dülger H., Dönder A., Sekeroğlu M.R., Erkoç R., Ozbay B. (2011). Investigation of the relationship between serum levels of cotinine and the renal function in active and passive smokers. Ren. Fail..

[10] Fu Y.C., Xu Z.L., Zhao M.Y., Xu K. (2022). The Association Between Smoking and Renal Function in People Over 20 Years Old. Front. Med..

[11] Jhee J.H., Joo Y.S., Kee Y.K., Jung S.Y., Park S., Yoon C.Y., Han S.H., Yoo T.H., Kang S.W., Park J.T. (2019). Secondhand Smoke and CKD. Clin. J. Am. Soc. Nephrol..

[12] Omoloja A., Jerry-Fluker J., Ng D.K., Abraham A.G., Furth S., Warady B.A., Mitsnefes M. (2013). Secondhand smoke exposure is associated with proteinuria in children with chronic kidney disease. Pediatr. Nephrol..

[13] Benowitz N.L. (1996). Cotinine as a biomarker of environmental tobacco smoke exposure. Epidemiol. Rev..

[14] Leonberg-Yoo A.K., Rudnick M.R. (2017). Tobacco Use: A Chronic Kidney Disease Accelerant. Am. J. Nephrol..

[15] Lang S.M., Schiffl H. (2024). Smoking status, cadmium, and chronic kidney disease. Ren. Replace. Ther..

[16] Xia J., Wang L., Ma Z., Zhong L., Wang Y., Gao Y., He L., Su X. (2017). Cigarette smoking and chronic kidney disease in the general population: A systematic review and meta-analysis of prospective cohort studies. Nephrol. Dial. Transpl..

[17] Jin A., Koh W.P., Chow K.Y., Yuan J.M., Jafar T.H. (2013). Smoking and risk of kidney failure in the Singapore Chinese health study. PLoS ONE.

[18] Lee S., Kang S., Joo Y.S., Lee C., Nam K.H., Yun H.R., Park J.T., Chang T.I., Yoo T.H., Kim S.W. (2021). Smoking, Smoking Cessation, and Progression of Chronic Kidney Disease: Results From KNOW-CKD Study. Nicotine Tob. Res..

[19] Tylicki L., Puttinger H., Rutkowski P., Rutkowski B., Horl W.H. (2006). Smoking as a risk factor for renal injury in essential hypertension. Nephron Clin. Pract..

[20] Yacoub R., Habib H., Lahdo A., Al Ali R., Varjabedian L., Atalla G., Kassis Akl N., Aldakheel S., Alahdab S., Albitar S. (2010). Association between smoking and chronic kidney disease: A case control study. BMC Public Health.

[21] Liao D., Ma L., Liu J., Fu P. (2019). Cigarette smoking as a risk factor for diabetic nephropathy: A systematic review and meta-analysis of prospective cohort studies. PLoS ONE.

[22] Wang S., Qin A., Pei G., Jiang Z., Dong L., Tan J., Tan L., Tang Y., Qin W. (2021). Cigarette smoking may accelerate the progression of IgA nephropathy. BMC Nephrol..

[23] Gul C.B., Yildiz A., Sag S., Oruc A., Ersoy A., Gullulu S. (2021). The Effect of Smoking on Endothelial Dysfunction in Autosomal Dominant Polycystic Kidney Disease Patients with Preserved Renal Function. Ren. Fail..

[24] Chen M., Ding N., Grams M.E., Matsushita K., Ishigami J. (2024). Cigarette Smoking and Risk of Hospitalization with Acute Kidney Injury: The Atherosclerosis Risk in Communities (ARIC) Study. Am. J. Kidney Dis..

[25] Liang K.V., Greene E.L., Oei L.S., Lewin M., Lager D., Sethi S. (2007). Nodular glomerulosclerosis: Renal lesions in chronic smokers mimic chronic thrombotic microangiopathy and hypertensive lesions. Am. J. Kidney Dis..

[26] Mickelsson M., Söderström E., Stefansson K., Andersson J., Söderberg S., Hultdin J. (2021). Smoking tobacco is associated with renal hyperfiltration. Scand. J. Clin. Lab. Investig..

[27] Maeda I., Hayashi T., Sato K.K., Koh H., Harita N., Nakamura Y., Endo G., Kambe H., Fukuda K. (2011). Cigarette smoking and the association with glomerular hyperfiltration and proteinuria in healthy middle-aged men. Clin. J. Am. Soc. Nephrol..

[28] Aissani M.S., Niskanen L., Tuomainen T.P., Ould Setti M. (2025). Renal Hyperfiltration as a New Mechanism of Smoking-Related Mortality. Nicotine Tob. Res..

[29] Yan L.J., Allen D.C. (2021). Cadmium-Induced Kidney Injury: Oxidative Damage as a Unifying Mechanism. Biomolecules.

[30] Jaimes E.A., Tian R.X., Raij L. (2007). Nicotine: The link between cigarette smoking and the progression of renal injury?. Am. J. Physiol. Heart Circ. Physiol..

[31] Van Laecke S., Van Biesen W. (2017). Smoking and chronic kidney disease: Seeing the signs through the smoke?. Nephrol. Dial. Transplant..

[32] Jensen K., Nizamutdinov D., Guerrier M., Afroze S., Dostal D., Glaser S. (2012). General mechanisms of nicotine-induced fibrogenesis. FASEB J..

[33] Arany I., Hall S., Reed D.K., Reed C.T., Dixit M. (2016). Nicotine Enhances High-Fat Diet-Induced Oxidative Stress in the Kidney. Nicotine Tob. Res..

[34] Rahman H.H., Niemann D., Munson-McGee S.H. (2022). Association of chronic kidney disease with exposure to polycyclic aromatic hydrocarbons in the US population. Environ. Sci. Pollut. Res. Int..

[35] Bozier J., Chivers E.K., Chapman D.G., Larcombe A.N., Bastian N.A., Masso-Silva J.A., Byun M.K., McDonald C.F., Crotty Alexander L.E., Ween M.P. (2020). The Evolving Landscape of e-Cigarettes: A Systematic Review of Recent Evidence. Chest.

[36] Kanobe M.N., Dull G.M., Darnell J., Jin T., Brown B., Coffield J., Keyser B.M., Fearon I.M., Makena P., Baxter S.A. (2024). Evaluation of environmental emissions from glo heated tobacco products and combustible Cigarettes. Environ. Adv..

[37] Gunduz I., Nordlund M., King J., Gustin B., Cudazzo G., Nesovic M., Butin Y., Stura E., Alriquet M., Chauhan M. (2025). A comparative assessment of HPHC yields and in vitro toxicity for 1R6F reference cigarette smoke versus aerosol generated by Tobacco Heating System 3.0. Aerosol Sci. Technol..

[38] Raja A., Zelikoff J.T., Jaimes E.A. (2022). A contemporary review of nephrotoxicity and e-cigarette use. Curr. Opin. Toxicol..

[39] Smith D.M., Christensen C., van Bemmel D., Borek N., Ambrose B., Erives G., Niaura R., Edwards K.C., Stanton C.A., Blount B.C. (2021). Exposure to Nicotine and Toxicants Among Dual Users of Tobacco Cigarettes and E-Cigarettes: Population Assessment of Tobacco and Health (PATH) Study, 2013–2014. Nicotine Tob. Res..

[40] Lee J.W., Kim Y., Kim Y., Yoo H., Kang H.T. (2020). Cigarette Smoking in Men and Women and Electronic Cigarette Smoking in Men are Associated with Higher Risk of Elevated Cadmium Level in the Blood. J. Korean Med. Sci..

[41] Lang S.M., Hoffmann J., Schiffl H. (2025). E-cigarettes and kidney health: Current knowledge and future perspectives. Int. Urol. Nephrol..

[42] Cancelada L., Sleiman M., Tang X., Russell M.L., Montesinos V.N., Litter M.I., Gundel L.A., Destaillats H. (2019). Heated Tobacco Products: Volatile Emissions and Their Predicted Impact on Indoor Air Quality. Environ. Sci. Technol..

[43] Leigh N.J., Palumbo M.N., Marino A.M., O’Connor R.J., Goniewicz M.L. (2018). Tobacco-specific nitrosamines (TSNA) in heated tobacco product IQOS. Tob. Control.

[44] Oakes J.M., Fuchs R.M., Gardner J.D., Lazartigues E., Yue X. (2018). Nicotine and the renin-angiotensin system. Am. J. Physiol. Regul. Integr. Comp. Physiol..

[45] Rohleder N., Kirschbaum C. (2006). The hypothalamic-pituitary-adrenal (HPA) axis in habitual smokers. Int. J. Psychophysiol..

[46] Scharf P., Rizzetto F., Xavier L.F., Farsky S.H.P. (2022). Xenobiotics Delivered by Electronic Nicotine Delivery Systems: Potential Cellular and Molecular Mechanisms on the Pathogenesis of Chronic Kidney Disease. Int. J. Mol. Sci..

[47] Singh G.B., Kshirasagar N., Patibandla S., Puchchakayala G., Koka S., Boini K.M. (2019). Nicotine instigates podocyte injury via NLRP3 inflammasomes activation. Aging.

[48] Jomova K., Alomar S.Y., Nepovimova E., Kuca K., Valko M. (2025). Heavy metals: Toxicity and human health effects. Arch. Toxicol..

[49] Ekong E.B., Jaar B.G., Weaver V.M. (2006). Lead-related nephrotoxicity: A review of the epidemiologic evidence. Kidney Int..

[50] Robles-Osorio M.L., Sabath-Silva E., Sabath E. (2015). Arsenic-mediated nephrotoxicity. Ren. Fail..

[51] Yin H., Li X., Wang C., Li X., Liu J. (2024). Nickel induces mitochondrial damage in renal cells in vitro and in vivo through its effects on mitochondrial biogenesis, fusion, and fission. Chem. Biol. Interact..

[52] Gopalakrishnan A.V. (2026). Lead-Induced Nephrotoxicity and Its Therapeutic Interventions: An Updated Review. Biol. Trace Elem. Res..

[53] Yang Q., Zuo Z., Zeng Y., Ouyang Y., Cui H., Deng H., Zhu Y., Deng J., Geng Y., Ouyang P. (2023). Autophagy-mediated ferroptosis involved in nickel-induced nephrotoxicity in the mice. Ecotoxicol. Environ. Saf..

[54] Li J., Dai X., Hu S., Yang Q., Jing Z., Zhou Y., Jian X. (2024). Nickel induces pyroptosis via the Nrf2/NLRP3 pathway in kidney of mice. Biol. Trace Elem. Res..

[55] Li S.Y., Tsai M.T., Kuo Y.M., Yang H.M., Tong Z.J., Cheng H.W., Lin C.C., Wang H.T. (2024). Aldehyde dehydrogenase 2 preserves kidney function by countering acrolein-induced metabolic and mitochondrial dysfunction. JCI Insight.

[56] İnci M., Zararsız İ., Davarcı M., Görür S. (2013). Toxic effects of formaldehyde on the urinary system. Turk. J. Urol..

[57] Zhan Z., Chen J., Zhou H., Hong X., Li L., Qin X., Fu H., Liu Y. (2024). Chronic alcohol consumption aggravates acute kidney injury through integrin β1/JNK signaling. Redox Biol..

